# YES, CHANGING HOW YOU TEACH DOES MAKE A DIFFERENCE

**DOI:** 10.1093/geroni/igz038.3244

**Published:** 2019-11-08

**Authors:** Edmund H Duthie, Kathryn Denson, Deborah Simpson, Steven Denson, Amanda Szymkowski

**Affiliations:** 1 Medical College of Wisconsin, Milwaukee, Wisconsin, United States; 2 Aurora UW Medical Group, Milwaukee, Wisconsin, United States

## Abstract

Perceptions of an educational experience’s value impact learning. “Hands-on” activities promote deeper learning and retention. Educators may jettison more poorly rated sessions, not having time for perceived content revisions based on evaluation data. We sought to determine if simply changing the sequence of a session’s activities, using the same content, improved learner evaluations. Using a session focused on application of resources for dementia patient caregivers, we provided two versions of the same content to 2 groups of clinicians. In session version #1 (V1), participants were asked about caregiver stresses and barriers and then viewed two video triggers of a dementia patient and a stressed family caregiver. Participants then identified the caregiver’s struggles and recommended resources. At the session’s end they were provided with a Geriatric Fast Fact (GFF) (www.geriatricfastfacts.com) that hyperlinked to a variety of evidence-based resources by topic. In session version #2 (V2), only the content was flipped. The GFF was presented prior to the video, with clinicians were then tasked to identify best resources using the GFF. The V2 cohort rated the session higher than V1 cohort on a 4-point scale (1= Excellent, 4= Poor). Overall quality of learning plan (V1 =1.4 ; V2 =1.3); Would you recommend the session to peers (V1 = 1.5; and V2 =1.2) and Overall course evaluation (V1 = 1.5; V2. = 1.4) all improved. Using learner evaluations to revise the sequence of the same content was an effective educational strategy. Don’t throw the baby out with the bathwater!

